# Trace of delirium after robotic lower abdominal tumor resection at different end-tidal carbon dioxide: a RCT trial

**DOI:** 10.1186/s12871-024-02617-3

**Published:** 2024-07-12

**Authors:** Jingwen Chen, Si Liang, Ming Wei, Yue Ma, Tianpeng Bi, Zheng Liu, Yang Song, Hong Chen, Yu Wang

**Affiliations:** 1 Department of Anesthesiology, Harbin Medical University Cancer Hospital, No. 150 Haping Rd., Nangang District, Harbin, 150081 China; 2 Department of Anesthesiology, Affiliated Hospital of He Bei University, Baoding, 071000 China; 3 Clinical Medical College, Hebei University, Baoding, 071000 China

**Keywords:** Postoperative delirium, End-tidal carbon dioxide, Robotic surgery, Tumor resection

## Abstract

**Background:**

Postoperative delirium (POD) often occurs in oncology patients, further increasing the medical and financial burden. Robotic technology in lower abdominal tumors resection reduces surgical trauma but increases risks such as carbon dioxide (CO_2_) absorption. This study aimed to investigate the differences in their occurrence of POD at different end-tidal CO_2_ levels.

**Method:**

This study was approved by the Ethics Committee of Affiliated Hospital of He Bei University (HDFY-LL-2022-169). The study was registered with the Chinese Clinical Trials Registry on URL: http://www.chictr.org.cn, Registry Number: ChiCTR2200056019 (Registry Date: 27/08/2022). In patients scheduled robotic lower abdominal tumor resection from September 1, 2022 to December 31, 2022, a comprehensive delirium assessment was performed three days postoperatively using the CAM scale with clinical review records. Intraoperative administration of different etCO_2_ was performed depending on the randomized grouping after intubation. Group L received lower level etCO_2_ management (31-40mmHg), and Group H maintained the higher level(41-50mmHg) during pneumoperitoneum. Data were analyzed using Pearson Chi-Square or Wilcoxon Rank Sum tests and multiple logistic regression. Preoperative mental status score, alcohol impairment score, nicotine dependence score, history of hypertension and diabetes, duration of surgery and worst pain score were included in the regression model along with basic patient information for covariate correction analysis.

**Results:**

Among the 103 enrolled patients, 19 (18.4%) developed postoperative delirium. The incidence of delirium in different etCO_2_ groups was 21.6% in Group L and 15.4% in Group H, respectively, with no statistical differences. In adjusted multivariate analysis, age and during of surgery were statistically significant predictors of postoperative delirium. The breath-hold test was significantly lower postoperatively, but no statistical differences were found between two groups.

**Conclusion:**

With robotic assistant, the incidence of postoperative delirium in patients undergoing lower abdominal tumor resection was not modified by different end-tidal carbon dioxide management, however, age and duration of surgery were positively associated risk factors.

## Introduction

Postoperative delirium (POD) is a fluctuating organic cerebral syndrome presents clinically with sharply differing confused state after surgery [1]. Disorders in mind and behavior have been known in various form, including psychomotor disturbances ranging from hypoactive to hyperactive subtypes, fluctuation from baseline attention and consciousness, change of the sleep–wake schedule, and the confusion in the perception or memory, etc. [2–4]. In a cohort study, nearly a third of cancer patients were estimated who accepted highly invasive surgery experienced POD [5]. The cost of POD is enormous, as the high medical and financial burden for patients [6–8]. Carbon dioxide (CO_2_), one of the crucial drivers in cerebral blood flow (CBF) regulations, has been used in MRI diagnosis [9]. CO_2_ alterations can lead to local paradoxical abnormalities in CBF [10]. In case intracranial blood steal occurs in behavioral and cognitive areas of the brain, we have to be alert for potential risk for POD.

In these contexts, end-tidal carbon dioxide(etCO_2_) has attracted attention for its potential utility in the prediction of POD in cancer patients. In this study, we aimed to investigate the association between POD and different levels of PetCO_2_ in this setting.

## Method

### Patients identification and exclusion

This was a single-center, randomized and double-blind trial, undertaken in a tertiary care hospital in China. Participants were recruited from patients with lower abdominal tumors scheduled to undergo robot-assisted cancer resection from September 1, 2022 to December 31, 2022. We randomly assigned participants to receive different respiratory management using etCO_2_ as an indicator.

The study enrolled patients aged over 18 years, and scheduled for elective robotic-assisted laparoscopic surgery (including colorectal, urinary and gynecological procedures), American Society of Anesthesiologists physical status of 1 to 3, patients were excluded if they meet any of the following: (a) Unexpected change of anesthesia: switch to inhalation anesthesia or intravenous-inhalational anesthesia; (b) Severe abnormity in end-tidal carbon dioxide; (c) Unexpected replacement of surgical technique: converted to laparotomy or conventional laparoscopy; (d) Patients with preoperative delirium or unable to fully participate in delirium screening, including blind, deaf, illiterate or communication handicapped.

### Randomization and blindness

After the screening survey based on the included and excluded standards, we use the online randomization tool, Research Randomizer (https://www.randomizer.org), to assign participants into two groups. The researchers use the tool to generate sets of half the sample size, in which unique and unsorted numbers with a range between 1 and 2 (representing the two groups) to keep 1:1 ratio. Group L received the lower level etCO_2_ management (31–40 mmHg), and Group H maintained the higher etCO_2_ level(41-50mmHg) during pneumoperitoneum. Clinicians and patients were blinded to the study intervention with screening from external view.

### Perioperative period

**Fig. 1:**
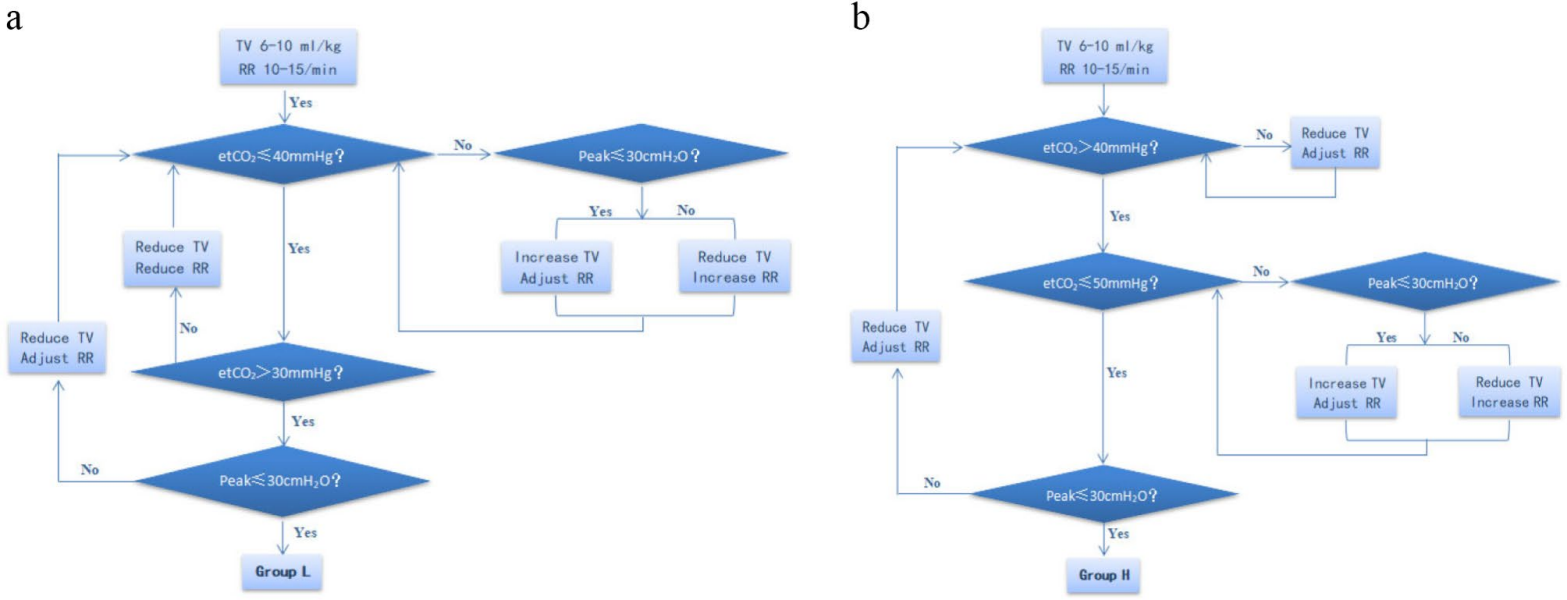
Respiratory management process. **a** End-tidal carbon dioxide adjustment process for Group L. **b** End-tidal carbon dioxide adjustment process for Group H. Abbreviations: TV, Tidal Volume; RR, Respiratory Rate; Peak, Peak Airway Pressure, etCO2, end-tidal carbon dioxide

All the patients were induced using etomidate, sufentanil, and cisatracurium, and total intravenous anesthesia was used with propofol, remifentanil and cisatracurium maintain appropriate anesthesia depth during surgery with bispectral index value 40–50. After the endotracheal tube inserted into the trachea, airway was managed with a mixture of oxygen and air, a tidal volume of 6-10 ml/kg predicted body weight, respiratory rate of 10–15/min, and PEEP of 1–3 cm H_2_O. Intraoperatively, the respiratory rate and tidal volume are continuously adjusted by closed-loop control mode to maintain the etCO_2_ target level while ensuring the peak airway pressure without over 30 cmH_2_O, as shown in Fig. 1.

Participants answered questions from the AUDIT-C [11], FTND [12] and MMSE [13] to quantify information about their alcohol use disorder, nicotine dependence and cognitive status before surgery. A visual analog scale evaluated their postoperative pain from 0 to 10, which indicates pain intensification [14]. The postoperative delirium, assessed by CAM scale combined with proximate retrospective medical and nursing notes in the preceding 24 h [15]. 

### Outcome and analysis

**Fig. 2:**
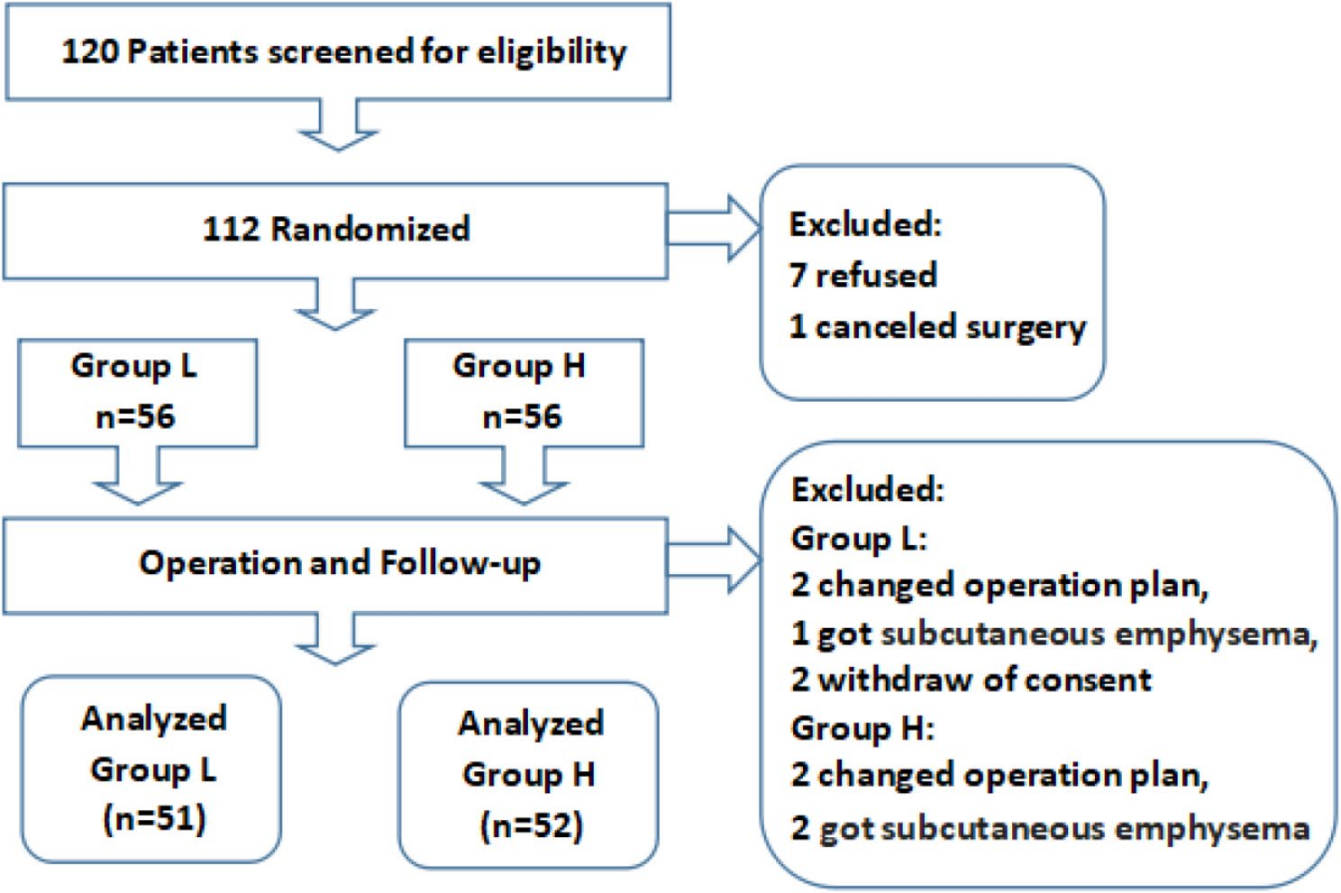
Patients flow diagram

Patients flow is shown in Fig. 2. Our primary outcome was the incidence of delirium at 3 days after surgery. The secondary outcomes were intraoperative circulation, bispectral index, postoperative pain, and changes in breath-hold test scores. Data were expressed, depend on their types and distribution, as mean ± standard deviation, median (IQR, interquartile range), or number (%, proportion). 2-tailed Chi-square test, t test and Mann-Whitney U-test were used for the statistical analysis as appropriate, also the logistic regression and adjusted models for covariates. Statistical analysis was completed using SPSS version 24.0 (SPSS, Inc.).

## Result

In all of the one hundred and three patients, nineteen patients developed POD, for an incidence of 18.4%. Fifteen (78.9% of patients with delirium) became delirious on postoperative day 1, four (21.1% of patients with delirium) became delirious on postoperative day 2, and four had POD symptoms present for postoperative day 1 to 3 (21.1% of patients with delirium).

**Fig. 3:**
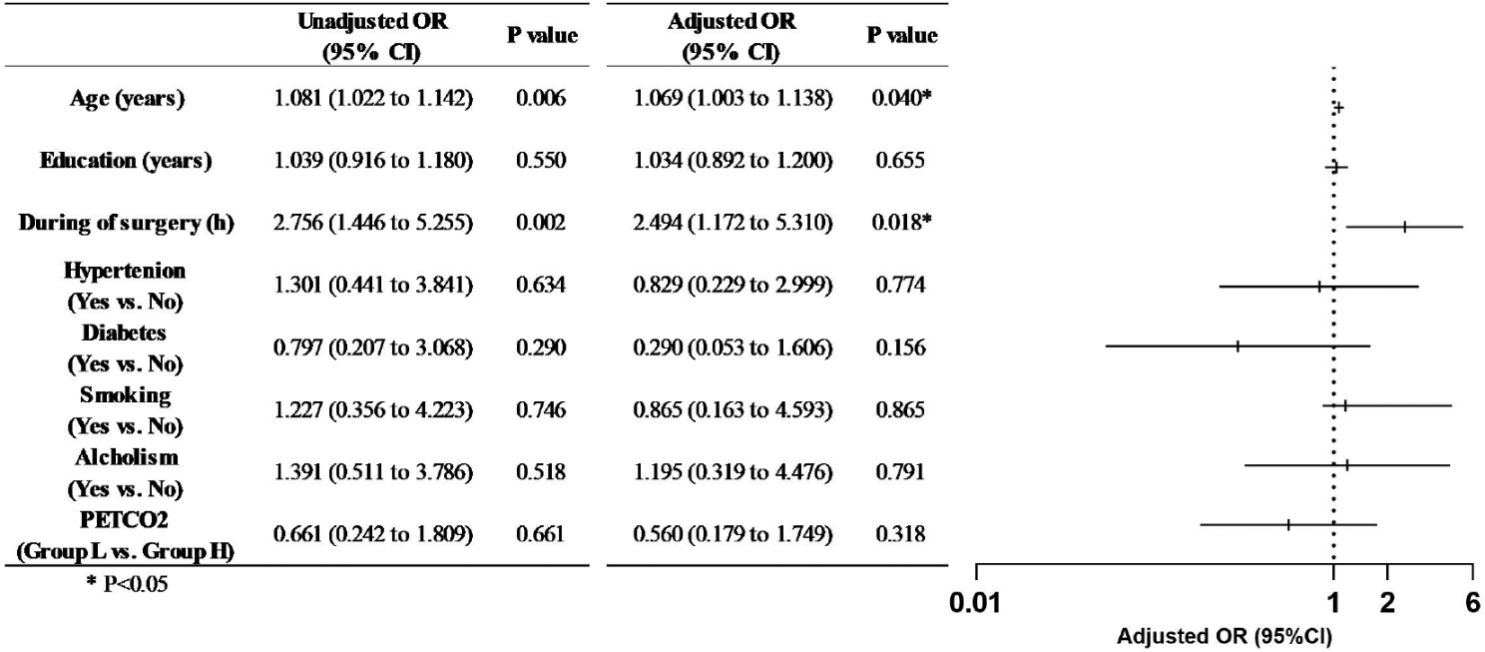
Univariate and multivariate associations with postoperative delirium

Overall clinical characteristics and baseline of patients (Table 1) were well balanced between the two groups. The incidence of POD in groups L and H were 21.6% (11/51) and 15.4% (8/52), respectively, and there were no differences in the two groups. No statistically significant associations were found in the univariate binary logistic regression between postoperative delirium and different the end-tidal partial pressure of CO_2_ (PetCO_2_) groups or other baseline medical conditions, except for age and during of surgery. In the adjusted multiple logistic tests, both age and during of surgery remained a statistically significant predictor of delirium (Fig. 3).

**Table 1:** Clinical characteristics of patients at baseline

Variables	Group L (*n* = 51)	Group H (*n* = 52)	*P* value
Age, mean ± SD(years)	59.0 ± 10.6	58.6 ± 11.0	0.857
BMI, median, IQR(kg/m^2^)^a^	24.5 (22.9–26.2)	25.2 (22.7–27.4)	0.391
Gender, No.(%)			
Male	23.0 (52.3)	21.0 (47.7)	0.629
Female	28.0 (47.5)	31.0 (52.5)	
ASA score, No.(%)			
I	0.0 (0.0)	3.0 (100.0)	0.450
II	17.0 (18.7)	74.0 (81.3)	
III	2.0 (22.2)	7.0 (77.8)	
Hypertension, No.(%)			
Yes	13.0 (46.4)	15.0 (53.6)	0.702
No	38.0 (50.7)	37.0 (49.3)	
Diabetes, No.(%)			
Yes	10.0 (52.6)	9.0 (47.4)	0.763
No	41.0 (48.8)	43.0 (51.2)	
Tumor site, No.(%)			
Colorectum	22.0 (47.8)	24.0 (52.2)	0.943
Urination	16.0 (50.0)	16.0 (50.0)	
Gynecology	13.0 (52.0)	12.0 (48.0)	
Education, median, IQR(years)	12.0 (9.0–12.0)	12.0 (9.0–15.0)	0.314
≤ Elementary School, No.(%)	10 (52.6)	9 (47.4)	0.569
Secondary School, No.(%)	30 (52.6)	27 (47.4)	
> Secondary School, No.(%)	11 (40.7)	16 (59.3)	
MMSE, median, IQR	28.0 (27.0–29.0)	28.0 (27.0–29.0)	0.688
FTND, median, IQR	0.0 (0.0–0.0)	0.0 (0.0–0.0)	0.804
AUDIT-C, median, IQR	0.0 (0.0–3.0)	0.0 (0.0–3.0)	0.823
Duration of surgery, mean ± SD(h)	2.7 ± 1.0	2.7 ± 0.8	0.914
Duration of mechanical ventilation, mean ± SD (h)	3.2 ± 1.0	3.2 ± 0.9	0.975

Abbreviations: IQR, interquartile range; BMI, Body Mass Index; MMSE, Mini Mental State Examination; FTND, Fagerström test for nicotine dependence; AUDIT-C, Alcohol Use Disorders Identification Test consumption questions

a Calculated as weight in kilograms divided by height in meters squared

We compared information on the occurrence of delirium under different management in two groups in Table 2. In the secondary outcome, the difference in the worst postoperative pain score was not statistically significant between the two etCO_2_ management modes, neither visual analog scale nor critical care pain observation tool score. No differences were found in heart rate and mean blood pressure monitoring 1 h after pneumoperitoneum.

**Table 2:** Primary and secondary outcomes

	PETCO2 Group, No./total, No.(%)^a^		*P* value
	Group L	Group H	
Primary outcome			
Postoperative delirium	11/51 (57.9)	8/52 (42.1)	0.419
Subtypes			
Hyperactive	3/19 (42.9)	4/19 (57.1)	0.466
Hypoactive	7/19 (63.6)	4/19 (36.4)	
Mixed motor agitation	1/19 (100.0)	0 (0.0)	
Onset time^b^			
Postoperative Day 1	10/19 (66.7)	5/19 (33.3)	0.262
Postoperative Day 2	1/19 (25.0)	3/19 (75.0)	
Secondary outcomes			
Circulation after 60 min of CO2 flow			
Heart Rate, median (IQR)	68 (60–76)	73 (63–78)	0.102
Mean Blood Pressure, median (IQR)	92 (80–105)	89 (80–103)	0.470
Worst pain score^d^			
VAS, median (IQR)	3 (2–5)	3 (2–5)	0.831
CPOT, median (IQR)^c^	1 (0–3)	1 (0–2)	0.685
Breath-holding test			
Preoperative BHT, median (IQR)	34 (25–44)	33 (25–44)	0.913
Postoperative Day 1, median (IQR)	20 (15–26)*	20 (15–25)*	0.869
Postoperative Day 2, median (IQR)	21 (15–32)*	20 (17–25)*	0.815
Postoperative Day 3, median (IQR)	23 (16–33)*	21 (16–28)*	0.313

*Compared to preoperative BHT of respective group, *p* < 0.001

Abbreviations: IQR, interquartile range; VAS, visual analog scale; CPOT, Critical Care Pain Observation Tool; BHT, breath holding time

a Values are reported as No./total No. (%) unless otherwise indicated

b Postoperative delirium onset time were defined as the date of first symptom appearance

c CPOT is based on four domains: Patient’s facial expressions, Body movements, Compliance with a ventilator (or voice use for non-intubated patients), Muscle tension

d Both VAS and CPOT score range was taken from the highest value (worst pain score) over 3 days

## Discussion

In this randomized, masking and controlled trial, we found 18.4% incidence of POD in adult patients undergoing elective lower abdominal tumors resection surgery with robots. Multivariate regression analysis highlighted age and duration of surgery as independent risk factors for delirium after robotic-assisted abdominal tumor resection. Various PetCO_2_ levels did not significantly change the incidence of postoperative delirium during the first 3 days, which were 21.6% and 15.4%, respectively.

etCO_2_ monitoring has been used in cardiopulmonary resuscitation and fluid resuscitation therapy widely [16, 17]. Apart from pulmonary blood flow, cardiac output and alveolar ventilation, it also has implications for perfusion of other organs, including cerebral perfusion, despite understudied field. Besides, as an important driver known to affect CBF, CO_2_ is physiologically relevant to cause changes in cerebrovascular activity [10]. It has been reported that decrease in etCO_2_ could cause a more vasoconstrictive response in functional areas related to executive ability, memory and cognition, such as the prefrontal cortex and hippocampus. These heterogeneous responses will contribute to the risk prediction of POD [18]. 

However, in studies of delirium after surgery procedures, scholars have found more irrelevance between POD and differential carbonic acidemia, which were consistent with our findings [19, 20]. In retrospective studies, conclusion that hypercapnia is a risk factor for POD has also been reported [21, 22]. Faced with this discrepancy between theory and clinic, [23–27] hypotheses have been proposed about the association between the duration of different levels of etCO_2_ or the magnitude of etCO_2_ variability and postoperative delirium [18, 28]. These hypotheses have been starting to find supporting evidence in the pathophysiology, but clinical studies are often limited by experimental conditions or cannot be effectively implemented due to patient safety concerns, which will provide direction for our future research.

We found that advanced age and long-time surgery are independent risk factors for POD. Increasing age, vascular elasticity decreases, blood perfusion becomes abnormal, and the risk of brain injury during surgery or anesthesia increases [29]. Increased intracerebral and plasma inflammatory factors (e.g., interleukin-6), reduced synapses in the brain and decreased mitochondrial function in the hippocampus can be observed [30]. 

Both innovation and limitations should be conceded. Firstly, we optimized the assessment of delirium as much as possible, but its diagnosis as a psycho behavioral state assessment tool remains much difficult. Secondly, neither hypertension nor diabetes in the participants of this trial was examined in more detail for cerebrovascular examination. Multicenter studies may yield richer findings. Thirdly,

our findings enrich the POD researches, and also create highly exploratory for future research due to the complexity of the mechanisms by which it occurs. Fourthly, the hypothesis is that POD will interact with changes in cerebral blood flow, so we need more data on the metabolic effects of CO_2_ replacement in the further study.

In conclusion, the incidence of postoperative delirium in patients undergoing lower abdominal tumor resection with robotic assistant was not modified by different end-tidal carbon dioxide management, however, age and duration of surgery were positively associated risk factors.

## Data Availability

All data generated or analyzed during this study are included in this published article.
